# Does selection of nostrils really affect performance of nasotracheal intubation with nasotracheal Airtraq®?

**DOI:** 10.3906/sag-1907-153

**Published:** 2020-02-13

**Authors:** Liu-Jia-Zi SHAO, Shao-Hua LIU, Fu-Shan XUE

**Affiliations:** 1 Department of Anesthesiology, Beijing Friendship Hospital, Capital Medical University, Beijing P.R. China

**To the Editor,**

In the recent article by Arslan and Türkyılmaz [1], comparing the performance of nasotracheal intubation (NTI) with the nasotracheal Airtraq® (Airtraq NT) between the right and left nostrils, they showed that NTI could be completed in a shorter time through the right nostril than through the left. Furthermore, both external laryngeal pressure and head flexion eased the NTI from the left nostril. These findings have potential implications for improvement of patient safety during NTI with the Airtraq NT, but we noted several issues in this study that might have influenced interpretation of the study results and we invite the authors to comment on these.

First, the readers were not provided with the details of NTI using the Airtraq NT. According to Figures 4 and 6 in the article, the Airtraq NT was inserted into the airway by a midline approach. It must be pointed out that the imaging channel of the Airtraq NT is at the left side of the blade [2]. When the midline insertion approach is used and the endotracheal tube is introduced through the right nostril, more room is allowed for manipulations of the Airtraq NT and endotracheal tube, and the use of Magill forceps on the right side of the oropharynx. However, the midline insertion approach can significantly affect the observation and manipulation of an endotracheal tube inserted into the left side of the oropharynx via the left nostril, and the use of Magill forceps. This may be a potential reason for more uses of auxiliary maneuvers, and a longer intubation time with the NTI through the left nostril in this study. Our experience suggests that when an endotracheal tube is inserted via the left nostril, the Airtraq NT should be inserted by a left approach, just like direct laryngoscopy. This can facilitate the NTI with the Airtraq NT via the left nostril [3].

Second, in this study, a cuffed spiral lateral beveled endotracheal tube was used for the NTI. Due to the lack of inherently anterior curvature, this endotracheal tube may often result in a posterior tube tip positioning. Thus, auxiliary maneuvers including the rotation of the endotracheal tube, external laryngeal pressure, cuff inflation, head flexion, and the use of Magill forceps are often required to direct the tube tip into the glottic opening. In contrast, with a good laryngeal exposure using the Airtraq NT, convenient or preformed PVC endotracheal tubes with inherently anterior curvature help to guide the tube tip into the glottic opening. Thus, if convenient or preformed PVC endotracheal tubes were used in this study, we argue that different results would have been obtained.

Third, in the materials and methods section, the authors described that when resistance was felt during the tube adjustment, the auxiliary maneuvers, including 90° counterclockwise rotation of the tube, external laryngeal pressure, cuff inflation, head flexion, changing the operator, and the use of Magill forceps, were applied in random order. It was unclear why these auxiliary maneuvers were used in random order. Such maneuvers should be selected based on the requirement for adjustment of inadequate tube tip positioning [4,5]. For example, external laryngeal pressure is suitable for a posterior tube tip positioning, head flexion for an anterior tube tip positioning, 90° counterclockwise rotation for a right tube tip positioning, and cuff inflation for a lateral or posterior tube tip positioning. In particular, we restate that the Airtraq NT provides additional space to facilitate passage of Magill forceps due to the absence of a tube-guiding channel, but a greater distal angulation of its blade may render the use of Magill forceps very awkward. Furthermore, the use of Magill forceps can result in a risk of cuff damage [3].

Finally, in this study, random selection of the right and left nostrils was used for NTI. Furthermore, both NTI time and total NTI time were regarded as important study endpoints. Because examination of the nasal cavity was not included in the airway characteristics of the patients, it was unclear whether the two groups were comparable with regard to the patency degree of the nostrils. It must be emphasized that anatomic aberrations of the nasal cavity are common; septal deviations and septal spurs together with turbinate hypertrophy often imply that one nasal cavity is more suitable for passage of an endotracheal tube than the other [6]. Thus, we are concerned that the unbalanced data of nasal anatomy would have biased the performance of NTI with the Airtraq NT.

We believe that addressing these above issues will further clarify the transparency of this study and avoid any optimistic interpretation or misinterpretation of study results.

**Authors’ contributions**

Liu-Jia-Zi Shao carefully read the paper of Arslan and Türkyılmaz, analyzed their data, suggested points of commentary, drafted this manuscript, and saw and approved the final manuscript. Shao-Hua Liu carefully read the manuscript of Arslan and Türkyılmaz, analyzed their methods and data, revised points of commentary and this manuscript, and saw and approved the final manuscript. Fu-Shan Xue carefully read the manuscript of Arslan and Türkyılmaz, analyzed their methods and data, revised the points of commentary and this manuscript, and saw and approved the final manuscript.
